# *PeVDE*, a violaxanthin de-epoxidase gene from moso bamboo, confers photoprotection ability in transgenic *Arabidopsis* under high light

**DOI:** 10.3389/fpls.2022.927949

**Published:** 2022-08-11

**Authors:** Yongfeng Lou, Huayu Sun, Chenglei Zhu, Kebin Yang, Xueping Li, Zhimin Gao

**Affiliations:** ^1^Key Laboratory of National Forestry and Grassland Administration/Beijing for Bamboo and Rattan Science and Technology, Institute of Gene Science and Industrialization for Bamboo and Rattan Resources, International Centre for Bamboo and Rattan, Beijing, China; ^2^Jiangxi Provincial Key Laboratory of Plant Biotechnology, Jiangxi Academy of Forestry, Nanchang, China

**Keywords:** *Phyllostachys edulis*, photoprotection, NPQ, *PeVDE*, violaxanthin cycle

## Abstract

Plants employ an array of photoprotection mechanisms to alleviate the harmful effects of high light intensity. The violaxanthin cycle, which is associated with non-photochemical quenching (NPQ), involves violaxanthin de-epoxidase (VDE), and zeaxanthin epoxidase (ZEP) and is one of the most rapid and efficient mechanisms protecting plants under high light intensity. Woody bamboo is a class of economically and ecologically important evergreen grass species widely distributed in tropical and subtropical areas. However, the function of VDE in bamboo has not yet been elucidated. In this study, we found that high light intensity increased NPQ and stimulated the de-epoxidation of violaxanthin cycle components in moso bamboo (*Phyllostachys edulis*), whereas, samples treated with the VDE inhibitor (dithiothreitol) exhibited lower NPQ capacity, suggesting that violaxanthin cycle plays an important role in the photoprotection of bamboo. Further analysis showed that not only high light intensity but also extreme temperatures (4 and 42°C) and drought stress upregulated the expression of *PeVDE* in bamboo leaves, indicating that *PeVDE* is induced by multiple abiotic stresses. Overexpression of *PeVDE* under the control of the *CaMV 35S* promoter in *Arabidopsis* mutant *npq1* mutant could rescue its NPQ, indicating that *PeVDE* functions in dissipating the excess absorbed light energy as thermal energy in bamboo. Moreover, compared with wild-type (Col-0) plants, the transgenic plants overexpressing *PeVDE* displayed enhanced photoprotection ability, higher NPQ capacity, slower decline in the maximum quantum yield of photosystem II (*F*_*v*_/*F*_*m*_) under high light intensity, and faster recovery under optimal conditions. These results suggest that *PeVDE* positively regulates the response to high light intensity in bamboo plants growing in the natural environment, which could improve their photoprotection ability through the violaxanthin cycle and NPQ.

## Introduction

Plants are challenged by various environmental stressors in nature, especially light stress. Although light is essential for photosynthesis, excessive light can cause oxidative damage to the photosynthetic apparatus by producing reactive oxygen species (ROS) (Müller et al., 2001; Takahashi and Badger, 2011). To prevent oxidative damage to the photosynthetic apparatus, plants have developed a series of photoprotection mechanisms (Goss and Lepetit, 2015; Leonelli et al., 2017; Velitchkova et al., 2020; Gray et al., 2022). One of the most rapid and efficient protection mechanisms is dissipating the excess absorbed light energy as thermal energy, which can be monitored as the non-photochemical quenching (NPQ) of chlorophyll fluorescence (Jahns and Holzwarth, 2012; Goss and Lepetit, 2015; Ruban and Wilson, 2021). NPQ consists of several components: energy quenching (qE), zeaxanthin-dependent NPQ (qZ), state transition (qT), photoinhibitory quenching (qI), sustained quenching (qH), and the fluorescence decay lacking component (qM) (Ruban et al., 2012; Ware et al., 2015; Malnoë, 2018; Pashayeva et al., 2021). As the main part of NPQ, qE is triggered by the generation of a pH gradient (ΔpH) across the thylakoid membrane and by two additional key factors: zeaxanthin (Z) and PsbS protein (Johnson et al., 2009; Ruban et al., 2012; Goss and Lepetit, 2015). In most plants, Z is a product of the violaxanthin cycle (also known as the xanthophyll cycle), and its accumulation is regulated by two enzymes, violaxanthin de-epoxidase (VDE) and zeaxanthin epoxidase (ZEP) (Goss and Jakob, 2010; Leonelli et al., 2016; Hoang et al., 2020; Lacour et al., 2020; Fernández-Marín et al., 2021).

The violaxanthin cycle exists in both higher plants and green algae and involves interconversions among three pigments, violaxanthin (V), antheraxanthin (A), and Z, which are catalyzed by VDE and ZEP (Hoang et al., 2020; Fernández-Marín et al., 2021). V is converted to Z *via* A by VDE under excessive light, and Z is converted back to V *via* A by ZEP under deficient light (Jahns et al., 2009; Li et al., 2016; Girolomoni et al., 2020; Goss and Latowski, 2020). The *npq1* and *npq2* mutants of *Arabidopsis thaliana* harbor mutations in the *VDE* and *ZEP* genes, respectively. These two mutants are defective in converting V to Z or constitutively accumulating Z; consequently, the ability to induce NPQ is either suppressed (*npq1*) or enhanced (*npq2*) (Niyogi et al., 1998; Ikeuchi et al., 2016). Overexpression of *Lycopersicon esculentum LeVDE* in tobacco could improve its tolerance to high light intensity and chilling stress by affecting NPQ (Han et al., 2010). Similar results were also obtained in transgenic *Arabidopsis* plants overexpressing *Cerasus humilis ChVDE* (Sun et al., 2019). In addition, NPQ was reduced by the overexpression of antisense *Cucumis sativus CsVDE* in *Arabidopsis* (Li et al., 2013). Therefore, VDE is indispensable for dissipating the excess absorbed light energy by NPQ through the violaxanthin cycle.

Bamboo is an ecologically and economically important group of plants and a renewable source of biomass, with great development potential and application prospects. More than 1,250 bamboo species have been identified to date (Benton, 2015), which account for 30 million hectares of forest area in the tropical, subtropical, and temperate regions. Bamboo forests are widely distributed, ranging from the sea horizon to an altitude of 4,000 m above sea level, and play an important role in developing regional economies and eliminating poverty. Unlike other crops, bamboos are a highly diverse group of plants of varying sizes, ranging from dwarf species (<1 m tall) to giant tropical species (up to 40 m in height) (Cao et al., 2012). Woody bamboo exhibits excellent properties such as high mechanical and tensile strength and high elasticity, which are highly beneficial for timber, artwork, and paper making applications. Because woody bamboo is an evergreen forest tree, with a vegetative phase generally lasting for more than a few decades, it must possess the ability to overcome multiple adverse environmental conditions, such as dehydration, chilling stress, and significant fluctuations in light intensity. Changes in light intensity can be caused by temporal and spatial differences in light environments, such as seasonal variation in sunlight duration and light scattering due to cloud movement, respectively. Although light is crucial for bamboo growth, photoprotection is indispensable for bamboo to adapt to different light environments. To adapt to the significant fluctuations in light intensity, bamboo species have evolved a series of sophisticated mechanisms that enable the harvesting and conversion of enough light energy for carboxylation while avoiding the formation of ROS. Moreover, bamboos are fast-growing plants, indicating that they possess strong photosynthetic capacity for carbon assimilation (Jiang et al., 2012).

To explore the molecular and regulatory mechanisms of photoprotection of bamboo, Zhao et al. (2016) performed RNA-seq analysis of moso bamboo (*Phyllostachys edulis*) leaves treated with high light intensity, which revealed some genes closely related to photoprotection, such as *PePsbS*, *PeVDE*, and *PeZEP*. The *PeZEP* and *PePsbS* genes of moso bamboo, which are related to NPQ, have been cloned and functionally characterized (Lou et al., 2017, 2018). The *PeVDE* gene of moso bamboo, which has also been cloned, is expressed primarily in leaves, and the encoded protein has been shown to convert V to Z through A *in vitro* (Gao et al., 2013). Although high light intensity has been shown to induce the expression level of *PeVDE*, it is still uncertain for the changes of A, V, and Z in moso bamboo leaves under high light intensity. Moreover, the relationship between *PeVDE* and NPQ *in vivo* is also unclear. The genome sequences of only six bamboo species have been published to date (Peng et al., 2013; Guo et al., 2019; Zheng et al., 2022), and the photoprotective molecular mechanism of bamboo has been explored less than the function of VDEs.

In the present study, to reveal the photoprotective function of *PeVDE* and the response of the violaxanthin cycle to high light intensity in bamboo, we determined NPQ and violaxanthin cycle components in the leaves of moso bamboo under high light intensity. Furthermore, we used VDE-deficient *npq1* mutant and wild-type *Arabidopsis* plants to analyze the function of *PeVDE* in moso bamboo.

## Materials and methods

### Plant materials, growth conditions, and treatments

Moso bamboo (*P. edulis*) seeds were sown and cultivated under laboratory conditions at 18–25°C, 16-h light/8-h dark photoperiod, and 250–350 μmol⋅m^–2^⋅s^–1^ light intensity. Six-month-old seedlings were used for different treatments. To conduct the varying light intensity treatment, 24-h dark-adapted seedlings were exposed to different light intensities (0, 300, 600, 900, 1,200, and 1,500 μmol⋅m^–2^⋅s^–1^), and leaf samples were collected after 2 h. To conduct high light intensity treatment, seedlings were exposed to 1,200 μmol⋅m^–2^⋅s^–1^ light intensity, and leaves were collected after 0, 1, 2, 3, 4, 8, and 12 h. To perform cold and heat treatments, seedlings were cultured at 4 or 42°C, respectively, for 0, 1, 2, 6, 12, and 24 h. In the drought treatment, watering was withheld for 17 days, and leaves were sampled at different time points during the drought period (0, 5, 7, 9, 11, 13, 15, and 17 days). To inhibit the VDE enzyme in bamboo leaves, detached leaves were incubated in distilled water containing 10 mM dithiothreitol (DTT) and dark-adapted for 6 h before measuring enzyme activity.

Seeds of *Arabidopsis thaliana* ecotype Columbia (Col-0; wild type [WT]) and mutant *npq1* (SALK_CS3771) were obtained from the Arabidopsis Biological Resource Center (ABRC; OH, United States), and sown on half-strength Murashige and Skoog (1/2 MS) medium or in peat moss and vermiculite mixture (1:1, v/v). Plants were grown in a growth chamber at 22°C under 16-h light/8-h dark photoperiod and fluorescent lamps with 150–250 μmol⋅m^–2^⋅s^–1^ light intensity.

### Measurement of violaxanthin xanthophyll component contents

To extract pigments, leaf samples were frozen in liquid nitrogen and ground using a mortar and pestle. Then, the ground leaf tissue was treated with 100% acetone, and the extract was gently mixed in the dark at 4°C for 1 h. Cell debris was removed by centrifugation two times at 12,000 rpm and 4°C for 10 min. The supernatant was passed through a 0.2-μm syringe filter. Pigment separation was performed in a high-performance liquid chromatography (HPLC) system (Gao et al., 2013). The de-epoxidation state (DES) of violaxanthin cycle components was calculated using the following equation:


DES=(A+Z)/(V+A+Z)

### Measurement of chlorophyll fluorescence

The chlorophyll fluorescence of photosystem II (PSII) of moso bamboo was measured *in vivo* using the actinic light model of IMAGING-PAM (Walz, Effeltrich, Germany). Prior to measurement, the plants were dark-adapted for 15 min. The maximum quantum yield of PSII (*F*_*v*_/*F*_*m*_) and NPQ was calculated as follows:


$$F_{V}/F\,m=\left(F_{m}-F_{o}\right)/F_{m}$$

$$N\,P\,Q=\left(F_{m}-F_{m}^{\prime }\right)/F_{m}^{\prime }$$

where *F*_*o*_ is the minimum fluorescence in the dark-adapted state; *F*_*m*_ is the maximum fluorescence in the dark-adapted state; and *F*_*m*_′ is the maximum fluorescence in any light-adapted state. *F*_*o*_, *F*_*m*_, and *F*_*m*_′ are obtained by applying saturating pulses. The chlorophyll fluorescence of *Arabidopsis* was also measured using this method.

### RNA extraction and gene expression analysis

Total RNA was extracted from all leaf samples using the TRIzol reagent (Invitrogen, Carlsbad, CA, USA), and treated with RNase-free DNase I (Promega, Madison, WI, USA) to remove any residual genomic DNA. Then, cDNA was synthesized from total RNA using the Reverse Transcription Kit (Promega, Madison, WI, USA), according to the manufacturer’s instructions.

Quantitative real-time PCR (qRT-PCR) was performed on a qTOWER 2.2 system (Analytik Jena, Germany) using the Roche Light Cycler 480 SYBR Green I Master kit. The qRT-PCR was performed in a 10.0-μL reaction volume containing 5.0 μL of 2 × SYBR Green I Master, 0.8 μL of cDNA, 0.2 μL of each primer (5.0 mM), and 3.8 μL of ddH_2_O. The thermocycling conditions were as follows: 95°C for 10 min, followed by 40 cycles of 95°C for 10 s and 60°C for 10 s. A 227-bp product of *PeVDE* was amplified using the PeVDE-qF/-qR primer pair. *PeNTB*, a nucleotide tract-binding protein gene of *P*. *edulis*, was amplified as a reference gene using the PeNTB-qF/-qR primer pair (Fan et al., 2013). Dissociation curve analysis of the amplification products was performed at the end of each PCR to confirm that only one PCR product was amplified and detected. The qRT-PCR was performed in three biological replicates for the reach gene. The relative expression level of *PeVDE* was calculated using the 2^–Δ^ ^Δ^ *^Ct^* method (Livak and Schmittgen, 2001). Primers used for qRT-PCR are listed in Supplementary Table 1.

### Vector construction and *Arabidopsis* transformation

To generate *VDE*-complementation and -overexpression lines, a *PeVDE* overexpression vector was constructed from pBI121 as described previously (Gao et al., 2013). Briefly, the open reading frame (ORF) of *PeVDE* was amplified from the cDNA of bamboo leaf samples using the primers PeVDE-F and PeVDE-R, which harbored *Bam*HI and *Sac*I restriction sites, respectively. The PCR product was cloned into the pBI121 vector downstream of the *CaMV 35S* promoter. The recombinant vector was introduced into *Agrobacterium tumefaciens* strain EHA105 by electroporation and then transformed into *Arabidopsis npq1* mutant and WT (Col-0) plants using the floral dipping method (Clough and Bent, 1998). T0 seeds were collected from the transgenic plants and selected on 1/2 MS medium containing 50 mg⋅L^–1^ of kanamycin. The identification of transgenic lines was validated by PCR. Homozygous T3 lines were used for subsequent experiments.

### High-light intensity treatment

Wild type (Col-0) seeds and homozygous T3 seeds of *PeVDE* overexpression lines were germinated on 1/2 MS medium and then transplanted into the peat moss: vermiculite mixture (1:1, v/v). After 4–8 weeks of growth, the seedlings were exposed to high light intensity (1,200 μmol⋅m^–2^⋅s^–1^) for 0, 1, 2, 3, and 4 h. Subsequently, the plants were transferred to darkness for recovery. The values of *F*_*v*_/*F*_*m*_ and NPQ were measured at each time point.

### Measurements of antioxidant enzyme activity and malondialdehyde content

After exposure to high light intensity, the activities of superoxide dismutase (SOD) and peroxidase (POD) enzymes and the content of malondialdehyde (MDA) in the leaves of transgenic and Col-0 plants were measured using the A001-1, A084-3, and A003-1 kits, respectively (Jiancheng, Nanjing, China), according to the manufacturer’s instructions.

### Histochemical staining of superoxide anion and hydrogen peroxide

Histochemical staining of superoxide anion (O_2_^•–^) and hydrogen peroxide (H_2_O_2_) was conducted as described previously (Zulfugarov et al., 2014), with some modifications. To detect O_2_^•–^, the leaf samples of transgenic and Col-0 plants were immersed in a 6 mM nitroblue tetrazolium (NBT) solution containing 50 mM sodium phosphate (pH 7.5), and incubated in the dark for 12 h. To detect H_2_O_2_ detection, the detached leaves were immersed in 5 mM 3, 3′-diaminobenzidine (DAB) solution containing 10 mM MES (pH 3.8) for 12 h under darkness. Subsequently, the NBT- and DAB-stained leaves were destained by boiling in lacto: glycerol:ethanol (1:1:4) solution for 5 min, and then cooled to room temperature before taking photographs.

### Statistical data analysis

A standard *t*-test was used to determine statistical significance with a 95% confidence interval. In all figures, data are represented as the mean ± standard error (SE).

## Results

### Kinetics of non-photochemical quenching induction in moso bamboo

**FIGURE 1:**
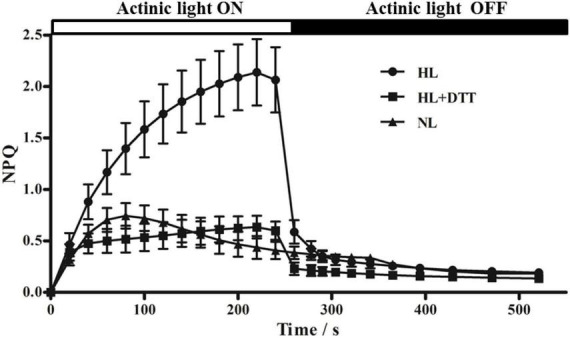
Non-photochemical quenching (NPQ) kinetics of chlorophyll fluorescence in moso bamboo. Samples were exposed to actinic light at two different intensities (230 μmol⋅m^–2^⋅s^–1^ [NL] and 1,075 μmol⋅m^–2^⋅s^–1^ [HL]) and then transferred to complete darkness. Additionally, one group of moso bamboo leaves (HL+DTT) was treated with DTT before being exposed to HL.

Non-photochemical quenching (NPQ) kinetics of chlorophyll fluorescence is deemed as a tool to reflect the NPQ processes. Figure 1 shows that actinic light induced an increase in NPQ in dark-adapted leaves; however, NPQ decreased to its original value when the actinic light was turned off. Under 230 μmol⋅m^–2^⋅s^–1^ actinic light, NPQ increased rapidly, reaching 0.75 at 80 s, and then gradually decreased to 0.40 at 240 s. However, under 1,075 μmol⋅m^–2^⋅s^–1^ actinic light, NPQ reached 1.40 at 80 s and 2.10 at 240 s, indicating that the higher NPQ showed more energy dissipation under high light intensity. After treatment with the VDE inhibitor (DTT, 10 mM), NPQ in leaves increased to 0.50 at 240 s under high light intensity conditions, which was significantly lower than the NPQ obtained in control leaves (no DTT treatment) (Figure 1). These data suggest that high light intensity induces energy dissipation in moso bamboo leaves, and the violaxanthin cycle might be responsible for NPQ under high light intensity.

We also analyzed the *F*_*v*_/*F*_*m*_ of DTT-treated and untreated leaves. No significant difference was detected in the *F*_*v*_/*F*_*m*_ of DTT-treated and untreated leaves under normal light intensity (Supplementary Figure 1). However, after exposure to high light intensity for 4 h, the value of *F*_*v*_/*F*_*m*_ declined to approximately 66.6 and 45.0% in leaves treated with and without DTT, respectively, which further suggests that VDE prevents photoinhibition *via* inducing energy dissipation in moso bamboo.

### Analysis of violaxanthin cycle components and non-photochemical quenching

**FIGURE 2:**
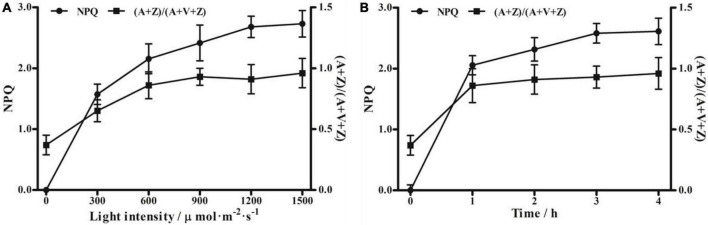
Non-photochemical quenching (NPQ) and de-epoxidation of the violaxanthin cycle in moso bamboo under different light conditions. **(A)** Treatment with different light intensities (0, 300, 600, 900, 1,200, and 1,500 μmol⋅m^–2^⋅s^–1^) for 2 h after dark adaptation for 24 h. **(B)** Treatment with high light intensity (1,200 μmol⋅m^–2^⋅s^–1^) for different times up to 4 h.

To determine the relationship between violaxanthin cycle components and NPQ capacity, moso bamboo seedlings were treated with different light intensities, and DES and NPQ were measured. The results showed that DES, which indicates Z-dependent thermal dissipation, was positively correlated with light intensity (Figure 2A). The value of DES increased from 0.37 in the dark condition to the maximum value of 0.96 under 1,500 μmol⋅m^–2^⋅s^–1^ light intensity. Additionally, the value of NPQ also increased with the increase in light intensity, from 0 in the dark to the maximum value of 2.73 under high light intensity (1,500 μmol⋅m^–2^⋅s^–1^). Under 1,200 μmol⋅m^–2^⋅s^–1^ light intensity, the DES increased rapidly to approximately 0.86 within the first hour, and then increased slowly, ultimately reaching a steady-state level over time. The value of NPQ also increased markedly in the first hour, and then increased slowly, reaching a peak of approximately 2.61 at the 4-h time point (Figure 2B). These results further demonstrate that violaxanthin cycle components play a critical role in NPQ in moso bamboo.

### Effects of variable light intensity on *PeVDE* expression

**FIGURE 3:**
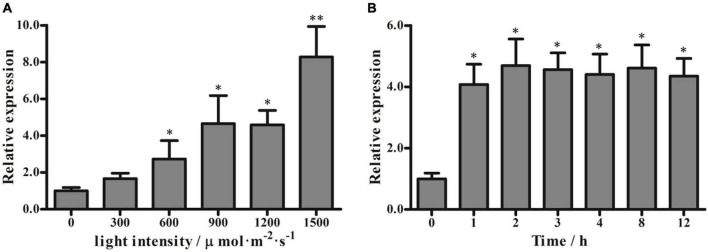
Transcript levels of *PeVDE* in moso bamboo leaves under different light conditions. **(A,B)** *PeVDE* expression in leaves treated with different light intensities (0, 300, 600, 900, 1,200, and 1,500 μmol⋅m^–2^⋅s^–1^) for 2 h **(A)**, and with high light intensity (1,200 μmol⋅m^–2^⋅s^–1^) for up to 12 h **(B)**. Asterisks indicate significant differences (*p < 0.05, ^**^p < 0.01).

To examine whether the expression of *PeVDE* is affected by light intensity, we examined the *PeVDE* transcript level in moso bamboo leaves exposed to different light intensities for 2 h. The results showed that the transcript level of *PeVDE* gradually increased with the increase in light intensity, reaching the highest level under 1,500 μmol⋅m^–2^⋅s^–1^ light intensity, which was 8.5-fold higher than that obtained in complete darkness (0 μmol⋅m^–2^⋅s^–1^) (Figure 3A). In addition, chlorophyll fluorescence analysis showed that *F*_*v*_/*F*_*m*_ decreased, whereas NPQ increased with the increase in light intensity (Supplementary Figure 2A). Furthermore, the light intensity of 1,200 μmol m^–2^⋅s^–1^ was sufficient to induce a significant stress response. To examine the expression of *PeVDE* in response to prolonged high light intensity stress, the moso bamboo seedlings were exposed to 1200 μmol⋅m^–2^⋅s^–1^ light intensity for up to 12 h. The transcript level of *PeVDE* significantly increased in the first hour, and then increased slowly, reaching a steady-state level, which was approximately 4.5-fold higher than that of the control (0 h) (Figure 3B). Additionally, NPQ increased, whereas *F*_*v*_/*F*_*m*_ decreased significantly in the first hour of high light stress; however, at subsequent time points, both NPQ and *F*_*v*_/*F*_*m*_ changed slowly and reached a relatively steady-state level with prolonged exposure to high light stress (Supplementary Figure 2B). These results indicate that *PeVDE* is a light-responsive gene, and NPQ is strongly correlated with the *PeVDE* transcript level in moso bamboo leaves.

### Effects of temperature extremes and drought stress on *PeVDE* expression

**FIGURE 4:**
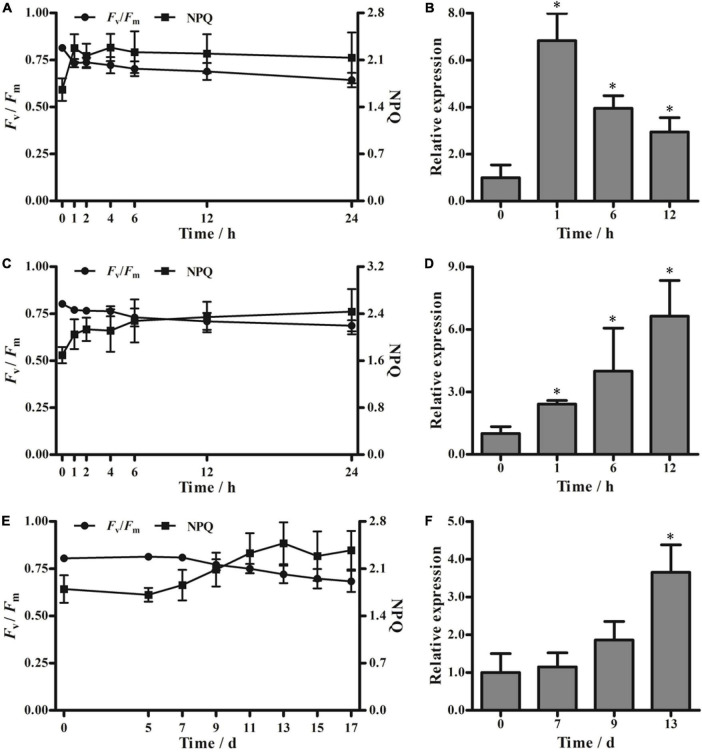
*F*_*v*_/*F*_*m*_, non-photochemical quenching (NPQ), and *PeVDE* transcript levels in moso bamboo leaves under different abiotic stresses. **(A–F)** Values of *F*_*v*_/*F*_*m*_ and NPQ **(A,C,E)** and relative transcript levels of *PeVDE* **(B,D,F)** in moso bamboo leaves treated with low-temperature stress (4°C) **(A,B)**, high-temperature stress (42°C) **(C,D)**, and drought stress **(E,F)**. NPQ was measured with the actinic light 530 μmol⋅m^–2^⋅s^–1^. Asterisks indicate significant differences (*p < 0.05).

Exposure to cold stress (4°C) and heat stress (42°C) altered the *F*_*v*_/*F*_*m*_ of moso bamboo seedlings. At both temperatures, *F*_*v*_/*F*_*m*_ decreased sharply within the first hour and then decreased slowly until the end of the stress treatment. Moreover, NPQ increased dramatically in the first hour and then displayed a slowly increasing trend during the remaining stress treatment (Figures 4A,C). To further investigate whether the low or high temperature stress could affect *PeVDE* expression, we selected samples collected at four representative time points, namely, 0, 1, 6, and 12 h. During low temperature stress, the abundance of *PeVDE* transcripts reached the maximum level in the first hour (approximately 6.8-fold higher than that at 0 h), and then started to drop gradually (Figure 4B). Unlike the low-temperature stress, high-temperature stress induced the expression of *PeVDE* continuously, which reached the maximum level at 12 h (approximately 6.6-fold higher than that at 0 h) (Figure 4D).

In the drought stress treatment, neither *F*_*v*_/*F*_*m*_ nor NPQ showed an abrupt change in the first 7 days (Figure 4E). Both *F*_*v*_/*F*_*m*_ and NPQ started to show obvious changes on the ninth day. Then, the *F*_*v*_/*F*_*m*_ decreased and NPQ increased, reaching a relatively steady-state level on the 13th day (Figure 4E). According to the changing trends of *F*_*v*_/*F*_*m*_ and NPQ under drought stress, samples collected at four representative time points (0, 7, 9, and 13 days) were selected for further investigation of the *PeVDE* transcript level. The expression of *PeVDE* increased gradually, reaching the maximum level on the 13th day (3.6-fold higher than that at 0 day) (Figure 4F). These results suggest that the expression of *PeVDE* is affected by low temperature, high temperature, and drought stresses.

### Functional complementation analysis of *PeVDE* in *npq1*

**FIGURE 5:**
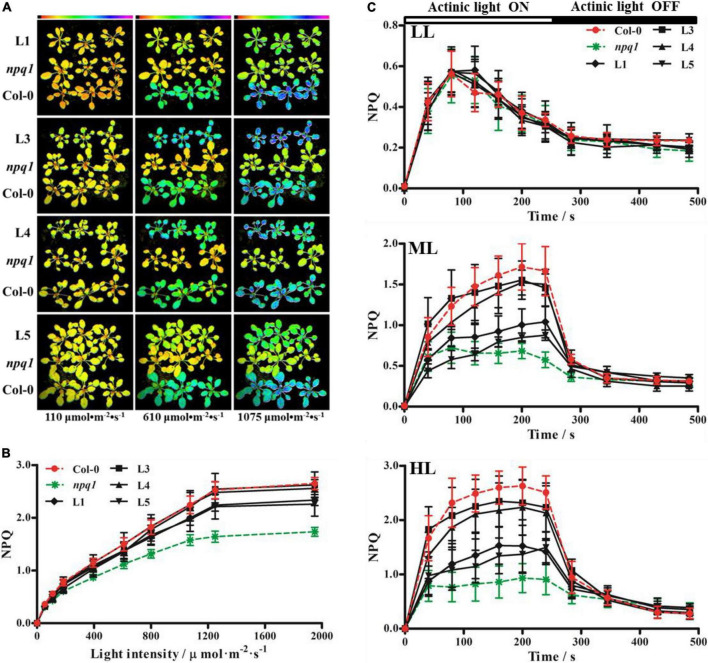
Non-photochemical quenching (NPQ) of Col-0, *npq1*, and *PeVDE* transgenic plants. **(A–C)** Chlorophyll fluorescence images **(A)**, NPQ **(B)**, and NPQ kinetics **(C)** of Col-0, *npq1*, and transgenic plants at different actinic light intensities. LL, 110 μmol⋅m^–2^⋅s^–1^; ML, 610 μmol⋅m^–2^⋅s^–1^; HL, 1,075 μmol⋅m^–2^⋅s^–1^.

A previous study indicated that *VDE* is a single-copy gene in *Arabidopsis* (Niyogi et al., 1998). The VDE-deficient *A. thaliana* mutant, *npq1*, is unable to convert V to Z. Hence, it is easy to differentiate the *npq1* mutant from Col-0 by monitoring NPQ. The vectors harboring *CaMV 35S*-driven *PeVDE* were constructed and transformed into the *npq1* mutant. The putative *PeVDE* transgenic plants were selected on 1/2 MS medium containing 50 mg ⋅L^–1^ of kanamycin, and the selected seedlings were further verified by PCR (Supplementary Figure 3). A total of four transgenic lines (L1, L3, L4, and L5) were randomly selected to perform further experiments. To investigate whether the ectopic expression of *PeVDE* could rescue the NPQ of the *npq1* mutant, the transgenic lines were analyzed under different actinic light conditions. Chlorophyll fluorescence imaging showed that NPQ values of the four transgenic lines were different from that of the *npq1* mutant, which was similar to that of Col-0, particularly under high light intensity (Figures 5A,B). NPQ kinetics of transgenic plants increased more than that of the *npq1* mutant. Furthermore, the absence of *VDE* was fully complemented in the transgenic lines L3 and L4 and partially complemented in lines L1 and L5 under medium and high light intensities (Figure 5C). Furthermore, the results of semi-quantitative RT-PCR revealed that *PeVDE* was expressed in all four transgenic lines (Supplementary Figure 3B), and the expression level of *PeVDE* in L3 and L4 was higher than that in L1 and L5. The expression level of *PeVDE* showed a positive correlation with NPQ under high light intensity. Considering all the above results, we concluded that the expression of *PeVDE* could rescue NPQ of *npq1*, and the Z synthesized by *PeVDE* performs the function of dissipating excess absorbed light energy as heat.

**FIGURE 6:**
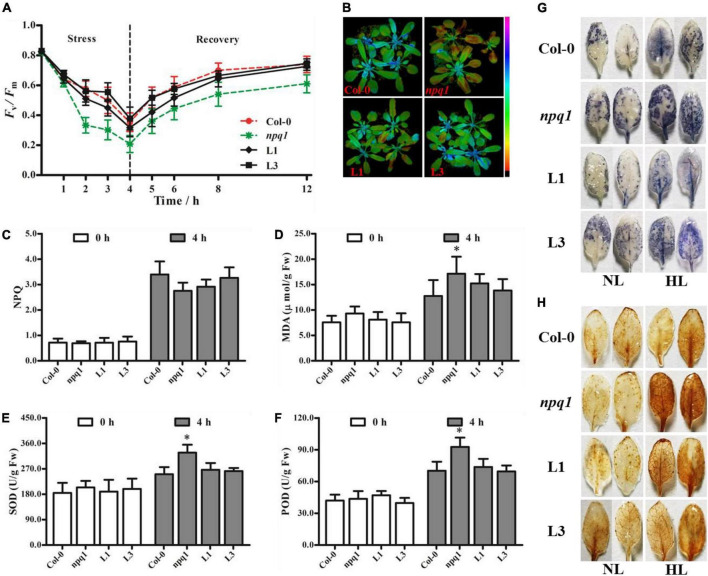
Overexpression of *PeVDE* in *npq1* mutant confers tolerance to high light intensity. **(A)** Quantification of *F*_*v*_/*F*_*m*_ under high light stress. **(B)** Chlorophyll fluorescence images of *F*_*v*_/*F*_*m*_. **(C)** NPQ. **(D)** MDA content. **(E)** SOD activity. **(F)** POD activity. **(G)** Representative images illustrating O_2_^•–^ accumulation in the leaves of Col-0, *npq1*, and transgenic lines were visualized by staining with NBT. **(H)** Representative images illustrating H_2_O_2_ accumulation in the leaves of Col-0, *npq1*, and transgenic lines, visualized by staining with DAB. NL, normal light; HL, high light. Asterisks indicate significant differences (*p < 0.05).

Based on these experiments, we selected two lines (L1 and L3) with different *PeVDE* expression levels for subsequent analyses. To assess the tolerance of Col-0, *npq1* mutant, and transgenic lines (L1 and L3) to high light intensity (1,200 μmol⋅m^–2^⋅s^–1^), we measured the *F*_*v*_/*F*_*m*_ of these genotypes. Before the exposure to high light intensity, no significant difference was detected in the *F*_*v*_/*F*_*m*_ values of the different genotypes (Figure 6A). However, after exposure to high light intensity, the *F*_*v*_/*F*_*m*_ decreased in all genotypes. After 4 h, the *F*_*v*_/*F*_*m*_ decreased markedly in the *npq1* mutant but only slightly in L1 and L3 compared with Col-0 (Figures 6A,B). After exposure to high light stress for 4 h, the plants were transferred to low light intensity (∼50 μmol⋅m^–2^⋅s^–1^), and a gradual recovery of *F*_*v*_/*F*_*m*_ was observed in all genotypes. The recovery rate of *F*_*v*_/*F*_*m*_ was better in Col-0, L1, and L3 plants than in the *npq1* mutant. After 8 h of recovery, the *F*_*v*_/*F*_*m*_ in L1 and L3 had returned to 87.6 and 88.4% of the initial *F*_*v*_/*F*_*m*_, respectively, and these recovery rates were close to that of Col-0 (89.8%); however, the *F*_*v*_/*F*_*m*_ of the *npq1* mutant returned only to 74.2% of the initial level (Figure 6A). In addition, the NPQ value of all genotypes increased rapidly, and the maximum NPQ was detected at the end of high light intensity stress. Although the NPQ values of L1, L3, and Col-0 showed no significant differences, they were all higher than the NPQ of the *npq1* mutant (Figure 6C). These results indicate that the overexpression of *PeVDE* could improve the NPQ and alleviate photoinhibition of PSII under high light intensity stress. Furthermore, histochemical staining analysis of Col-0, *npq1* mutant, L1, and L3 leaves before high light intensity treatment revealed no significant differences in O_2_^•–^ and H_2_O_2_ levels, and the level of O_2_^•–^ was lower than that of Col-0. After high light intensity stress, O_2_^•–^ accumulations in the leaves of all genotypes increased. The intensity of dark blue color was stronger in *npq1* mutant leaves than in Col-0, L1, and L3 leaves (Figure 6G). Because O_2_^•–^ is rapidly converted into the more stable compound (H_2_O_2_) by SOD, we monitored H_2_O_2_ accumulation by histochemical staining with DAB. Because of O_2_^•–^ accumulation, the accumulation of H_2_O_2_ in *npq1* mutant leaves was greater than that in Col-0 and transgenic plants under high light stress (Figure 6H). These results suggest that *PeVDE* participates in photoprotection by reducing the accumulation of O_2_^•–^ and H_2_O_2_.

Malondialdehyde is an indicator of membrane lipid peroxidation. Before high light stress, no significant difference in the MDA content was observed among Col-0, *npq1* mutant, and transgenic plants. After high light intensity stress for 4 h, a remarkable increase in MDA content was noted in all samples. The MDA content of *npq1* increased more quickly and significantly than that of transgenic and Col-0 plants (Figure 6D), indicating that membrane damage was greater in *npq1* than in Col-0 and transgenic plants under high light stress. The activity of ROS-scavenging enzymes (SOD and POD) also was examined. Compared with Col-0 and transgenic plants, the *npq1* mutant showed higher activity of SOD and POD (Figures 6E,F). This result suggests that *PeVDE* plays a critical role in reducing the accumulation of ROS under high light intensity stress.

### Overexpression of *PeVDE* in wild type *Arabidopsis* plants

Since the overexpression of *PeVDE* could rescue the *npq1* mutant phenotype by increasing the NPQ, we overexpressed *PeVDE* under the control of the *CaMV 35S* promoter in WT *Arabidopsis* (Col-0) plants to verify whether PeVDE could enhance the ability of photoprotection. A total of nine independent transgenic lines were obtained, among which three lines (L7, L8, and L9) were further confirmed by PCR (Supplementary Figure 4) and used to investigate the ability of photoprotection.

**FIGURE 7:**
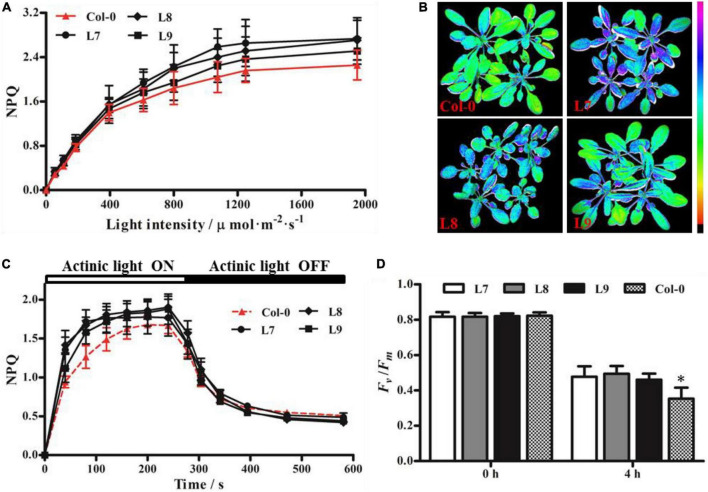
Chlorophyll fluorescence parameters of *Arabidopsis* (Col-0) plants overexpressing *PeVDE*. **(A)** NPQ of Col-0 and transgenic plants at different light intensities. **(B)** Chlorophyll fluorescence images of NPQ in Col-0 and transgenic plants at high light intensity (1,250 μmol⋅m^–2^⋅s^–1^). **(C)** NPQ kinetics of Col-0 and transgenic plants at 925 μmol⋅m^–2^⋅s^–1^ actinic light. **(D)** Values of *F*_*v*_/*F*_*m*_ under high light stress. Asterisks indicate significant differences (*p < 0.05).

Distinguishing between transgenic and Col-0 plants based on NPQ was difficult under low to moderate light intensities; however, the NPQ of transgenic plants was higher than that of Col-0 under high light intensity (Figure 7A). Furthermore, we performed chlorophyll fluorescence imaging (Figure 7B) and NPQ analysis (Figure 7C) under high light intensity (1,250 μmol⋅m^–2^⋅s^–1^) and moderate light intensity (925 μmol⋅m^–2^⋅s^–1^), respectively. The results showed that all transgenic plants had higher NPQ capacity than Col-0. After exposure to high light stress for 4 h, the *F*_*v*_/*F*_*m*_ of transgenic plants was higher than that of Col-0 plants (Figure 7D). These results indicate that *PeVDE* overexpression enhances the ability of NPQ under high light intensity and alleviates the photoinhibition of PSII in transgenic plants.

## Discussion

High light intensity is typical stress found in nature that induces the photoinhibition of PSII and causes photodamage to the photosynthetic apparatus (Takahashi and Badger, 2011; Ruban, 2016). To avoid photoinhibition and photodamage, plants have developed diverse photoprotection mechanisms, such as light avoidance associated with the movement of leaves and chloroplasts; screening of photo radiation; ROS scavenging systems; thermal energy dissipation; cyclic electron flow around PSI; and the photorespiratory pathway. Thermal dissipation can be quantified by measuring the NPQ of chlorophyll fluorescence, which is one of the most studied topics in photoprotection research (Takahashi and Badger, 2011). One of the important factors inducing NPQ in plants and green algae is the violaxanthin cycle. Previous studies showed that NPQ is positively correlated with the DES of V (Goss and Lepetit, 2015; Li et al., 2016). In the current study, we found that high light intensity increased the NPQ and stimulated the de-epoxidation of V and A in moso bamboo, whereas treatment with the VDE inhibitor (DTT) decreased the NPQ capacity of leaves (Figure 1), indicating that *PeVDE* plays an important role in the process of photoprotection, consistent with the results found in green algae (Mou et al., 2013; Zhang et al., 2013). Additionally, DES and NPQ showed similar trends, which increased with the increase in light intensity and rapidly increased in the first hour under high light intensity (Figure 2). This result is consistent with the results of previous studies on *Cucumis sativus*, *Ulva prolifera*, and *Panax notoginseng* (Li et al., 2013; Mou et al., 2013; Zhang et al., 2021), which further supports the importance of *PeVDE* in photoprotection. These data suggest that the violaxanthin cycle plays a critical role in the NPQ of moso bamboo. We also found that violaxanthin cycle pigment conversion and NPQ kinetics in moso bamboo were quicker to reach stable status, with lower light intensity (∼900 μmol⋅m^–2^⋅s^–1^) and shorter induction time (∼1 h), than those in tomato and cucumber (Han et al., 2010; Li et al., 2013). This indicates that the *VDE* gene of moso bamboo responds to high light intensity stress more quickly and directly than the *VDE* genes of other crops.

Violaxanthin de-epoxidase is a key enzyme in the violaxanthin cycle that catalyzes the conversion of V to Z through A under high light intensity. The regulation of VDE could take place at the transcriptional, post-transcriptional, or translational level. In plants, *VDE* genes have been shown to positively or negatively regulate the tolerance to abiotic stresses, especially high light intensity stress (Wang et al., 2022). The *PeVDE* gene was cloned from moso bamboo and was shown to be primarily expressed in leaves (Gao et al., 2013). In the present study, we investigated the expression of *PeVDE* under different light intensities and found that *PeVDE* was upregulated with the increase in light intensity. Moreover, the expression level of *PeVDE* increased rapidly in the first hour under high light intensity, and then continued to increase slowly over time (Figure 3). This suggests that *PeVDE* is a light-inducible gene. Moreover, NPQ also increased with the increase in light intensity (Supplementary Figure 2), which indicates that *PeVDE* is responsible for the increase in NPQ in moso bamboo in response to changes in light intensity. Similar results were reported in *C. sativus* and *Lycium chinense* (Li et al., 2013; Guan et al., 2015). In addition, we also analyzed the expression of *PeVDE* in moso bamboo leaves under other environmental stresses that could induce photoinhibition (Figure 4). The results showed that the expression of *PeVDE* was induced by low temperature, high temperature, and drought stress, consistent with the results of *CsVDE*, *LcVDE*, and *AhVDE* expression analyses (Li et al., 2013; Guan et al., 2015; Yang et al., 2015).

Because the field of the molecular biology of bamboo started relatively late, studies on bamboo genetics are challenged by factors such as an unpredictable and extremely long flowering cycle, plant death after flowering, low seed setting rate, and lack of a genetic transformation system. Therefore, we had to perform part of the functional analysis of *PeVDE* in *Arabidopsis*. Overexpression of *PeVDE* could rescue the NPQ ability of the *Arabidopsis* mutant *npq1* (Figure 5), indicating that *PeVDE* performs the same function as *AtVDE*. This result is in agreement with the earlier studies, suggesting that VDE exhibits a conserved function in plants (Xu et al., 2016). At the same time, our results supported the previous results that the protein encoded by *PeVDE* contains three conserved domains (Cys-rich, lipocalin, and Glu-rich), and could catalyze the conversion of V to Z through A *in vitro* (Gao et al., 2013). The violaxanthin cycle-related NPQ is a very important mechanism that protects PSII under high light intensity (Goss and Lepetit, 2015). Previous studies suggested that the *VDE* gene plays an important role in NPQ; overexpression and silencing of the *VDE* gene can change the NPQ ability and subsequently affect the sensitivity of PSII to high light intensity (Gao et al., 2010; Han et al., 2010; Yang et al., 2015). A similar result was also obtained in the present study. Overexpression of *PeVDE* in the *npq1* mutant or Col-0 could reduce the sensitivity to photoinhibition under high light intensity by enhancing the ability of NPQ (Figures 6, 7). Excessive light energy leads to the accumulation of ROS to harmful levels (Takahashi and Badger, 2011), and more ROS are produced in the chloroplasts when NPQ is inhibited (Zulfugarov et al., 2014). In the present study, histochemical staining of *npq1* mutant leaves showed that the overexpression of *PeVDE* could reduce the accumulation of O_2_^•–^ and H_2_O_2_ under high light intensity stress, probably because NPQ could dissipate the excess absorbed light energy and Z could scavenge the ROS directly (Demmig-Adams et al., 2020). In short, overexpression of *PeVDE* could not only rescue the photoprotection ability of the *npq1* mutant but also increase the photoprotection ability of transgenic Col-0 plants, consistent with previous studies (Guan et al., 2015).

## Conclusion

The current study reports the expression features of *PeVDE* in bamboo under abiotic stress, especially under high light intensity. The elevated expression of *PeVDE* improved bamboo tolerance to stress by driving the violaxanthin cycle and increasing the DES. Overexpression of *PeVDE* could rescue the NPQ of the *Arabidopsis npq1* mutant, indicating that *PeVDE* functions in dissipating the excess absorbed light energy as thermal energy in bamboo. Chlorophyll fluorescence analysis could reflect the changes in NPQ equally well in both moso bamboo and *Arabidopsis*, consistent with the expression of *PeVDE*. These results indicate that the Z synthesized by *PeVDE* plays an important role in avoiding photoinhibition and improving the ability of photoprotection in moso bamboo. Nonetheless, further research is needed to validate the function of *PeVDE* in bamboo directly.

## Data availability statement

The original contributions presented in this study are included in the article/Supplementary material, further inquiries can be directed to the corresponding author.

## Author contributions

ZG designed and supervised this study. YL and HS performed the experiments and collected all the data. CZ, KY, and XL provided a method for the data analysis. YL and ZG prepared the manuscript. HS and ZG modified the manuscript. All authors contributed to the article and approved the submitted version.
