# Supplementary material for: PeVDE, a violaxanthin de-epoxidase gene from moso bamboo, confers photoprotection ability in transgenic Arabidopsis under high light
Source: Front Plant Sci. 2022 Aug 11;13:927949. doi: 10.3389/fpls.2022.927949 (PMC9403991; doi:10.3389/fpls.2022.927949)
Supplement: Supplementary file 1 [file Data_Sheet_1.docx]

Supplementary Material

# Supplementary Figures and Tables

## Supplementary Figures

**Supplementary Figure 1.** Changes in the *F*_v_/*F*_m_ of moso bamboo leaves under different light conditions. **(A)** Chlorophyll fluorescence images of *F*_v_/*F*_m_. **(B)** Quantification of *F*_v_/*F*_m_. LL, 230 μmol·m^-2^·s^-1^; HL, 1075 μmol·m^-2^·s^-1^; HL+DTT, 1075 μmol·m^-2^·s^-1^ + DTT-treated. Asterisks indicate significant differences (**p* < 0.05, ** *p* < 0.01).

**Supplementary Figure 2.** Changes in the *F*_v_/*F*_m_ and NPQ of moso bamboo leaves under different light conditions. **(A, B)** Variation in *F*_v_/*F*_m_ and NPQ under different light intensities (0, 300, 600, 900, 1200, and 1500 μmol·m^-2^·s^-1^) for 2 h **(A)** and under high light intensity stress (1200 μmol·m^-2^·s^-1^) for up to 12 h **(B)**.

**Supplementary Figure 3.** Validation of *PeVDE* transgenic *Arabidopsis* (*npq1*) plants. **(A)** PCR analysis of genomic DNA. **(B)** Expression analysis of *PeVDE* in *Arabidopsis* plants by RT-PCR. M, DNA marker DL5000; L1, L3, L4, and L5, transgenic lines; Col-0, wild-type *Arabidopsis*; *npq1*, *Arabidopsis* mutant *npq1*; P, positive control (plasmid); d, negative control (ddH_2_O).

**Supplementary Figure 4.** Validation of *PeVDE*-overexpressing *Arabidopsis* (Col-0) plants. **(A)** PCR analysis of genomic DNA. **(B)** Expression analysis of *PeVDE* in *Arabidopsis* plants by RT-PCR. M, DNA marker DL5000; L7, L8, and L9, transgenic lines; Col-0, wild-type *Arabidopsis*; P, positive control (plasmid); d, negative control (ddH_2_O).

**Supplementary Table 1.** List of primers used in this study.

| Primer name | Nucleotide sequence (5′→3′) | Application |
| --- | --- | --- |
| PeVDE-qF | CCTCGACTCAGAGTGTTCCCTC | *PeVDE* expression analysis |
| PeVDE-qR | GGTAACCTTGACATGGAGGTGATC |  |
| PeVDE-F | GCGGATCCATGATGTCGCGGCAGTGCG | *PeVDE* expression vector construction |
| PeVDE-R | CGGAGCTCCTACCTTAGCTTCCTTATTGGCAGGGA |  |
| PeNTB-qF | TCTTGTTTGACACCGAAGAGGAG | Reference gene |
| PeNTB-qR | AATAGCTGTCCCTGGAGGAGTTT |  |
| AtUbi-F | ATGGAAAATCCCACCTACTAAATT | Reference gene |
| AtUbi-R | TTGAACAACTCGTAGCAACTCATC |  |
